# A Novel Necroptosis-Related miRNA Signature for Predicting the Prognosis of Breast Cancer Metastasis

**DOI:** 10.1155/2022/3391878

**Published:** 2022-03-25

**Authors:** Lin Zheng, Jie Wang, Hongnan Jiang, Honglin Dong

**Affiliations:** ^1^Department of Vascular Surgery, The Second Hospital of Shanxi Medical University, Taiyuan, China; ^2^Department of Breast Surgery, The Second Hospital of Shanxi Medical University, Taiyuan, China

## Abstract

**Objective:**

Necroptosis was recently identified as a form of programmed cell death that plays an essential role in breast cancer metastasis. MicroRNAs (miRNAs) have long been recognized to affect cell death and tumor growth. In this study, we aimed to screen for necroptosis-associated miRNAs that predict breast cancer metastasis.

**Method:**

This study used The Cancer Genome Atlas (TCGA) public database to obtain miRNA expression data and associated clinical data from breast cancer patients and then retrieved miRNA data related to necrosis and apoptosis. Next, using Cox regression model analysis (univariate or multivariate) as well as a comparison analysis (differential analysis), a prognostic multi-miRNA molecular marker was established. Finally, prognosis-related miRNAs were utilized to identify target genes, and the functions of the target genes were analyzed for enrichment to investigate the probable mechanisms of the miRNAs.

**Results:**

Ten miRNAs were screened through differential analysis to build models: hsa-miR-148a-3p, hsa-miR-223-3p, hsa-miR-331-3p, has-miR-181a-5p, hsa-miR-181b-5p, hsa-miR-181c-5p, hsa-miR-181d-5p, hsa-miR-200a-5p, hsa-miR-141-3p, and hsa-miR-425-5p. The multivariate Cox regression model was an independent prognostic factor (univariate Cox regression results: HR = 3.2642, 95%CI = 1.5773 − 6.7554, *P* = 0.0014; multivariate Cox regression results: HR = 3.1578, 95%CI = 1.5083 − 6, *P* = 0.0023). The survival curve of the risk score also revealed that patients with a high risk score had a poor prognosis (*P* = 2e − 04). The receiver operating characteristic (ROC) curve showed that the model has a certain prediction ability. Batch survival analysis of the miRNAs in the model was conducted and showed that hsa-miR-331-3p (*P* = 0.0182) was strongly associated with prognosis. Twenty-three predicted target genes were obtained, and Gene Ontology (GO) enrichment analysis showed that these target genes were strongly enriched in transcriptional initiation and cell membrane trafficking.

**Conclusion:**

Our research identified a novel miRNA marker for predicting breast cancer patient prognosis and lays the groundwork for future research on necroptosis-related genes.

## 1. Introduction

Breast cancer (BC) is the most common cancer and the fifth leading cause of cancer deaths worldwide [1]. Although the mortality rate of BC has decreased due to the development of treatment methods such as surgery, radiation, chemotherapy, endocrine therapy, and targeted therapy, systemic treatment to prevent metastasis is less effective [2]. Metastatic disease remains the underlying cause of death in the majority of BC patients [3]. Therefore, it is crucial to understand the mechanisms behind the metastatic process and to identify effective therapeutic targets and prognostic biomarkers for BC. Necroptosis is a recently discovered cell death pattern independent of caspase that differs from apoptosis and necrosis. It is mediated by death receptors such as receptor-interacting protein kinase (RIP) 1, TNF receptor 1, and RIP3, which activate the phosphorylation of mixed lineage kinase domain-like (MLKL) protein, causing the cells to lose their integrity [4, 5]. Necroptosis is being investigated as a possible cancer therapy because of its important role in cancer development [6]. There have been two roles identified for necroptosis in cancer: first, one or a mix of necroptosis regulators may enhance cancer metastasis and progression; second, necroptosis can act as “insurance,” preventing tumor formation and metastasis when apoptosis is damaged [7, 8]. According to the reports, an increasing number of medications and substances induce necroptosis to combat cancer [9–11]. Intracellular signaling proteins such as pattern recognition receptors (PRRs), the tumor necrosis factor receptor (TNFR) superfamily, T cell receptors (TCRs), and several chemotherapeutic medications have been discovered to play a role in necroptosis. Important regulators include RIP1 and RIP3. Necrostatin-1 (Nec-1) may also prevent necroptosis specifically [7]. However, there have been few findings on necroptosis signaling mediated by noncoding RNAs (ncRNAs).

Similar to siRNAs, microRNAs (miRNAs) are small molecules generated by advanced eukaryote genomes [12]. The miRNA-guided silencing complex (RISC) degrades or blocks the translation of the target gene mRNA by base matching with it [13]. miRNAs as suppressors of gene expression, have the ability to control more than 30% of mRNAs, and are involved in the development, apoptosis, cell proliferation, cell differentiation, and stress response [14]. Through a variety of pathways, the acquisition and loss of miRNA function promote cancer growth [15]. Higher levels of circulating miR-122 are associated with BC metastasis. MiR-122 secreted by cancer cells inhibits the glucose uptake of cells in the premetastatic niche by reducing the activity of the glycolytic enzyme pyruvate kinase, thereby promoting disease progression [16]. Although several studies have examined the role of miRNAs in the onset and progression of breast carcinoma, none has examined the use of necroptosis-related miRNAs to predict BC patient prognosis. As a result, it is still unclear whether necroptosis-related miRNAs are linked to patient prognosis; therefore, further research on molecular markers for predicting the prognosis of BC patients utilizing necroptosis-related miRNAs is needed.

To solve the above problem, this study first retrieved the miRNA expression data and related clinical data of BC patients from The Cancer Genome Atlas (TCGA) database and extracted the miRNA data related to necrosis and apoptosis. Then, a prognostic multi-miRNA molecular marker was constructed by differential analysis and univariate and multivariate Cox regression analyses. Finally, the target genes were predicted using miRNAs associated with prognosis, and the functional enrichment analysis of these genes was carried out to investigate the possible mechanisms of these miRNAs.

## 2. Materials and Methods

### 2.1. Data Collection

This study downloaded the miRNA expression data and relevant clinical information of BC patients from the TCGA database, including the number of samples of miRNA data and the number of patients with clinical data. In this study, we collected cancer metastasis-related miRNAs regulating cell necroptosis from the literature [17] and then extracted the expression matrix of negative apoptosis-related miRNAs for data matching filtering and correction, as well as for filtering and matching relevant clinical data for subsequent analysis.

### 2.2. Construction of a Prognostic Model of Necrotic Apoptosis-Related miRNAs

In this study, the R software package limma was used to analyze the differences in the processed data. The filtering conditions were as follows: the absolute value of log_2_ fold change (log_2_FC) was greater than 0, and the false discovery rate (FDR) was <0.05. The necroptosis-related miRNAs that met the above filtering conditions were considered to be differentially expressed. The acquired miRNAs related to the differentially expressed necroptosis-related genes were examined using batch univariate Cox regression analysis, based on the results of univariate Cox regression analysis. To build a multivariate Cox regression model and calculate the risk score, the miRNAs highly related to necroptosis were chosen. In addition, the risk score obtained by the model was combined with clinical factors for univariate Cox regression analysis and multivariate Cox regression analyses. We determined whether the risk score was an independent predictor based on the results of the Cox regression analyses. In this study, the prediction ability of the model was evaluated by drawing receiver operating characteristic (ROC) curves and calculating the areas under the curve (AUCs). The low-risk and high-risk groups were divided according to the median value of the risk score, and a risk-related survival curve was constructed. Finally, the survival curve of miRNAs in the model were drawn in batches to further clarify the miRNAs related to BC patient prognosis.

### 2.3. Enrichment and Analysis of miRNA Target Genes Related to Necroptosis

The target genes of necroptosis-associated miRNAs that were strongly linked to prognosis are predicted using miRDB, TargetScan, and miRTarBase. The target genes commonly identified in these three databases were selected as the target genes of the necroptosis-related miRNAs. Cytoscape software was used to construct the miRNA target gene network, and the R package “clusterProfiler” was used to perform Gene Ontology (GO) and Kyoto Encyclopedia of Genes and Genomes (KEGG) [18].

## 3. Results

### 3.1. Result of Necroptosis-Associated miRNA Prognostic Model

**Figure 1:**
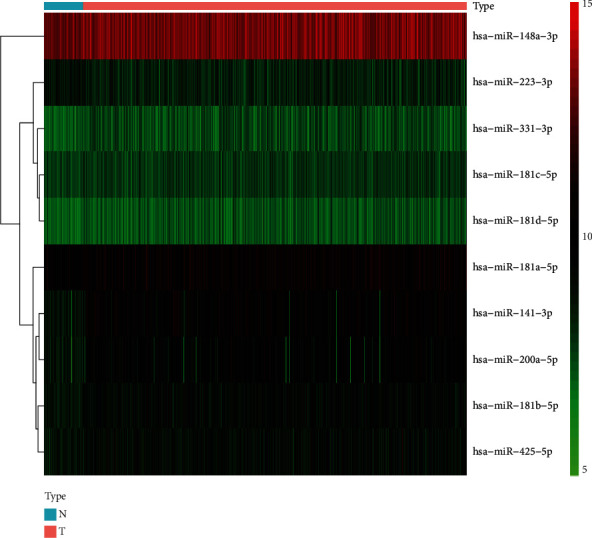
The heat map of 10 miRNAs differentially expressed between normal (N) and tumor (T) tissues. Red represents tumor tissue. Blue represents normal tissue.

**Figure 2:**
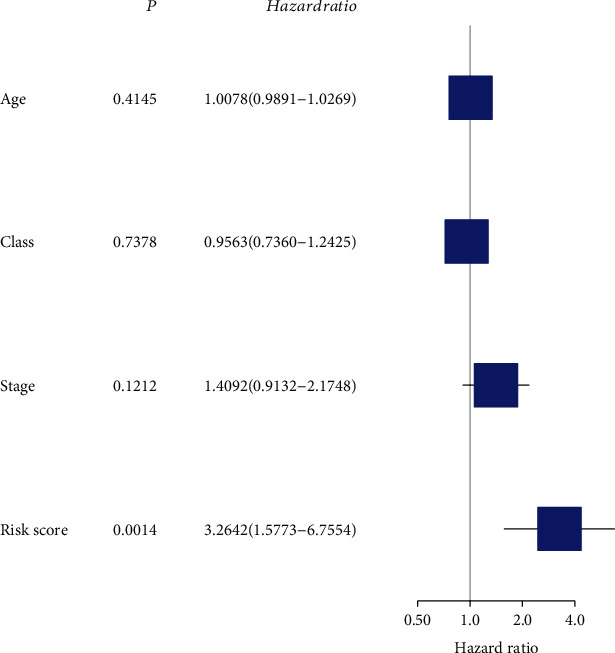
Univariate Cox analysis to identify risk factors.

**Figure 3:**
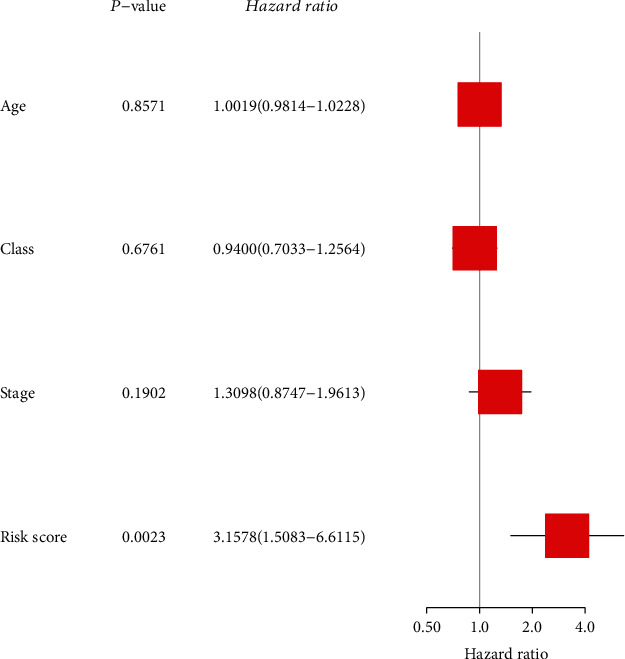
Multivariate Cox analysis to identify risk factors.

**Figure 4:**
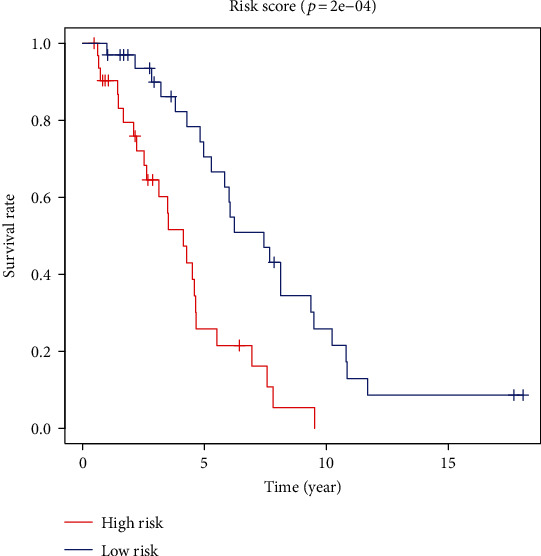
Overall survival analysis of risk score.

**Figure 5:**
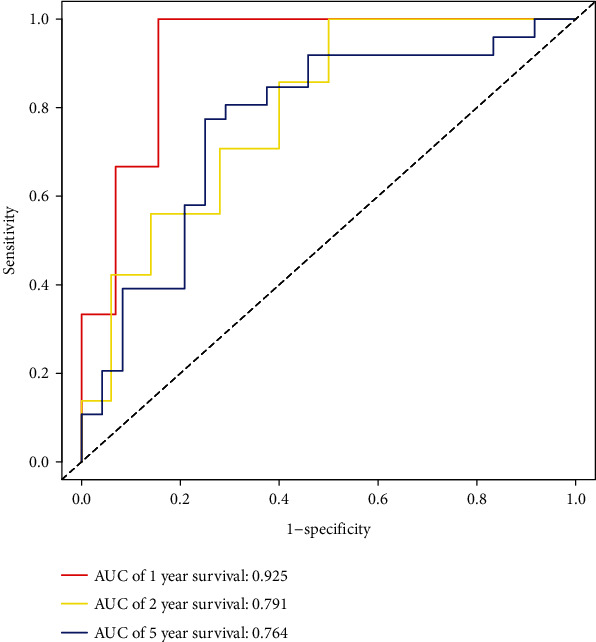
ROC curves results.

**Figure 6:**
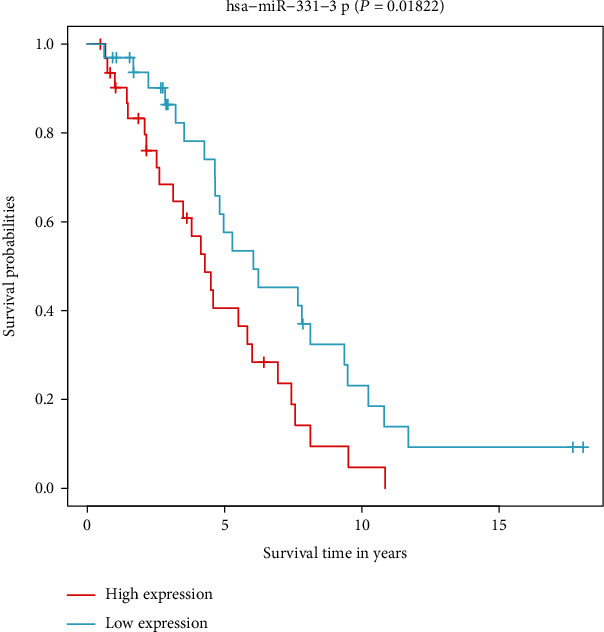
Overall survival analysis of hsa-miR-331-3p.

According to the results of the differential analysis, a total of ten different miRNAs were obtained: hsa-miR-148a-3p, hsa-miR-223-3p, hsa-miR-331-3p, hsa-miR-181a-5p, hsa-miR-181b-5p, hsa-miR-181c-5p, hsa-miR-181d-5p, hsa-miR-200a-5p, hsa-miR-141-3p, and hsa-miR-425-5p (Figure 1). These miRNAs were abnormally expressed in BC tissues compared with normal tissues. These results showed that these differentially expressed necroptosis-related miRNAs are worthy of in-depth study. Therefore, we selected the above ten miRNAs to construct a multivariate Cox regression model, calculated the risk score, and then combined the risk score with clinical factors for univariate and multivariate Cox regression analyses. The results show that the risk score of this model was an independent prognostic factor. The worse the prognosis is, the greater the risk score (univariate Cox regression results: hazard ratio (HR): 3.2642, 95% confidence interval (CI): 1.5773-6.7554, *P* = 0.0014, Figure 2; multivariate Cox regression results: HR: 3.1578, 95% CI: 1.5083-6.6115, *P* = 0.0023, Figure 3). In addition, the survival curve of risk score revealed that BC patients with a high risk score had a poor prognosis (P =2e-04, Figure 4). The results of ROC curve show that the model has a certain prediction ability (Figure 5). To further understand the link between the prognosis of BC patients and the miRNAs related to necroptosis, batch survival analysis was performed on the miRNAs in the model. We discovered that hsa-miR-331p-3p (*P* = 0.0182) was substantially associated with the prognosis of the BC. The higher its expression is, the worse the prognosis (Figure 6).

### 3.2. Enrichment Analysis Results of Target Genes

**Figure 7:**
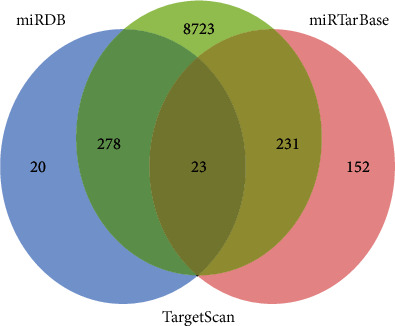
Venn diagram of the intersection of hsa-miR-331-3p predicted target genes from three miRNA databases: TargetScan, miRDB, and miRTarBase.

**Figure 8:**
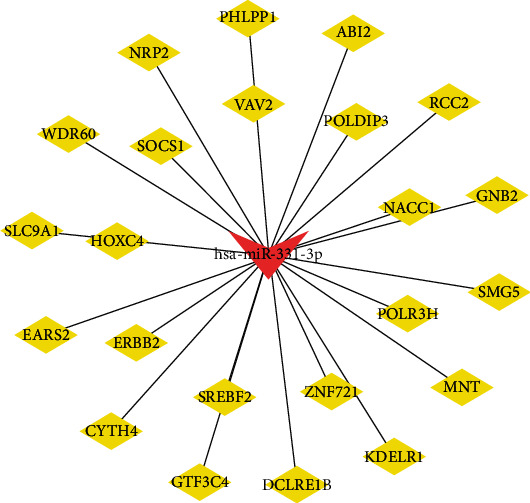
Network of hsa-miR-331-3p-target genes.

**Figure 9:**
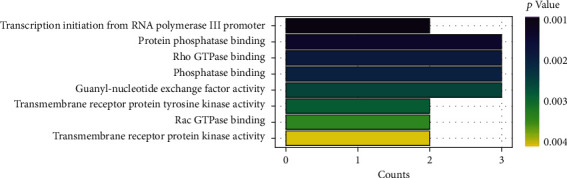
Gene Ontology (GO) enrichment analysis of the 23 target genes of hsa-miR-331-3p.

We selected the miRNA hsa-miR-331-3p, which was associated with a better prognosis in BC to predict target genes, and 23 target genes were obtained (Figure 7). The interaction network between hsa-miR-331-3p and its target genes was constructed by Cytoscape software (Figure 8). Finally, we performed GO enrichment analysis of the 23 target genes. The results of GO enrichment analysis showed that these target genes were significantly enriched in the following: transcription initiation from the RNA polymerase III promoter, protein phosphatase binding, Rho GTPase binding, phosphatase binding, guanyl-nucleotide exchange factor activity, transmembrane receptor protein tyrosine kinase activity, Rac GTPase binding, and transmembrane receptor protein kinase activity (Figure 9). This indicates that hsa-miR-331-3p is likely to be related to these functions and pathways.

The clusterProfiler R package was used to identify GO terms. A darker color means that the enriched pathways are more significantly different. Count refers to the number of genes in a certain pathway.

## 4. Discussion

BC, as the most common malignant tumor in females worldwide, poses a serious threat to the life and health of women and has become a key global public health problem. To improve the understanding of the development mechanism, an increasing number of studies have made efforts to find the regulator molecules. There are many studies on molecular markers using miRNAs to forecast the outcome of BC patients. For example, miR-186-5p and miR-548c-3p promote cell migration and invasion in BC by regulating the expression of the C-X-C motif chemokine receptor 4 (CXCR4), thereby affecting the survival rates of patients with triple-negative BC (TNBC) [19]. Additionally, regarding apoptosis in BC, miR-326 accelerates the cell cycle by targeting fibroblast growth factor receptor-1 (FGFR1). However, no study has systematically investigated the use of necroptosis-related miRNAs as molecular markers to forecast the outcome of BC patients. To the best of our knowledge, this study is the first to explore the use of necroptosis-related miRNAs in BC prognostic prediction.

In this study, the following necroptosis-related miRNAs linked to cancer metastasis were collected from the literature: miR-495, miR-331-3p, miR-15a, miR-148a-3p, miR-7-5p, miR-141-3p, miR-425-5p, miR-200a-5p, miR-210, miR-223-3p, miR-500a-3p, miR-181-5p, and miR-16-5p. Then, ten miRNAs were screened through differential analysis to build models (hsa-miR-148a-3p, hsa-miR-223-3p, hsa-miR-331-3p, hsa-miR-181a-5p, hsa-miR-181b-5p, hsa-miR-181c-5p, hsa-miR-181d-5p, hsa-miR-200a-5p, hsa-miR-141-3p, and hsa-miR-425-5p). An independent molecular marker for tumors was discovered using our approach. Hsa-miR-331-3p has a substantial correlation with BC survival, and it may be linked to the incidence, progression, and metastasis of BC.

It has recently been established that necroptosis is a kind of cell death that is preprogrammed and is mediated by the necroptosis molecules RIPK1, RIPK3, and MLKL [7]. It is similar to apoptosis in mechanism and to necrosis in morphology. This form of death has the contradictory effect of antitumor and tumor promotion. In the present study, we found that miRNAs related to necroptosis have prognostic function in cancer patients. For has-miR-331-3p, SNHG20 leads to the activation of HER2 in tumors by interacting with it, enhancing tumor cell invasion and migration [20]. Therefore, it can be speculated that SNHG20 coexpressed with has-miR-331-3p was associated with promoting cancer development by necroptosis. Regarding miR-148a-3p, reduced levels of its expression have been shown to reduce osteosarcoma cell death. Furthermore, lncRNA-107053293 modulates chicken tracheal cell necroptosis by serving as a ceRNA of hsa-miR-148a-3p [21, 22]. In addition, we noticed that in animals and cells, high concentrations of hsa-miR-200a-5p promoted RIP3-induced necroptosis [23]. RIP1 can play a critical role in necroptosis when it forms a complex with RIP3 [24]. Moreover, the combination of carboplatin and exosomes produced from the KRAS gene led to RIP3-/TNF*α*-mediated necroptosis in chemoresistant tumors in distal-tumor patients with hsa-miR-146/hsa-miR-210 regulation [25]. Rats were induced to develop acute kidney injury (AKI) by 3-MCPD-dipalmitate. Then, hsa-miR-223-3p was considerably enhanced leading to RIPK3 reduction by combining with the 3′ untranslated region of RIPK3 [26]. Atrazine promoted necroptosis in carp lymphocytes by downregulating miR-181-5p and activating the immune system, as well as increasing glycolysis [27]. hsa-miR-141-3p acted on RIPK1 and reduced necroptosis of intestinal endothelial cells that were treated with LPS [28]. hsa-miR-425-5p has been demonstrated to decrease the necroptosis mediated by combining with RIP1 and reducing RIP1 directly. The development of necroptosis was reduced by hsa-miR-425-5p, which reduced the inflammatory response and acute liver damage [29]. Furthermore, since hsa-miR-425 boosted MLKL phosphorylation by targeting RIPK1 transcripts, hsa-miR-425-5p depletion was connected to the cellular mechanism of Parkinson's disease [30]. Our study comprehensively analyzed the relationships among necroptosis, miRNAs, and the prognosis of BC, which has a certain significance for innovation and provides accuracy of clinical prognostic predictions. However, in this study, we used data from only the TCGA public database to build the model and were unable to collect their own clinical data to verify the model, which is a limitation of our study. In addition, we did not perform experiments to verify the expression, function, and action mechanism of these miRNAs, which requires further experimental exploration.

## 5. Conclusion

In conclusion, our study shows that necroptosis is closely related to BC, because there is a significant difference in miRNA expression between cancerous and noncancerous breast tissues. In addition, our model of five necroptosis-related miRNAs can be used as a significant predictor of BC. New miRNA markers for BC prognosis were identified in our work, and this offers a foundation for the discovery of further necroptosis-related genes in the future.

## Data Availability

The data used to support the findings of this study are included within the article.

## Abbreviations

ncRNA: Noncoding RNA
miRNA: MicroRNA
RISC: miRNA-guided silencing complex
TCRs: T cell receptors
TNFR: Tumor necrosis factor receptor
RIP1: Receptor-interacting protein kinase 1
RIP3: Receptor-interacting protein kinase 3
GO: Gene Ontology
KEGG: Kyoto Encyclopedia of Genes and Genomes.
